# Sarcopenia Prevalence and Risk Factors among Residents in Aged Care †

**DOI:** 10.3390/nu14091837

**Published:** 2022-04-28

**Authors:** Phillipa Darroch, Wendy J. O’Brien, Hajar Mazahery, Carol Wham

**Affiliations:** School of Sport, Exercise and Nutrition, College of Health, Massey University, Auckland 0745, New Zealand; phillipadarroch@gmail.com (P.D.); w.j.obrien@massey.ac.nz (W.J.O.); h.mazahery@massey.ac.nz (H.M.)

**Keywords:** sarcopenia, aged care, malnutrition, EWGSOP

## Abstract

The aim of this study was to investigate the prevalence of sarcopenia and associated risk factors among older adults living in three residential aged care (RAC) facilities within Auckland, New Zealand. A total of 91 older adults (63% women, mean age ± SD; 86.0 ± 8.3 years) were recruited. Using the European Working Group on Sarcopenia in Older People criteria, sarcopenia was diagnosed from the assessment of: appendicular skeletal muscle mass/height^2^, using an InBody S10 body composition analyser and a SECA portable stadiometer or ulna length to estimate standing height; grip strength using a JAMAR handheld dynamometer; and physical performance with a 2.4-m gait speed test. Malnutrition risk was assessed using the Mini Nutrition Assessment–Short Form (MNA-SF). Most (83%) of residents were malnourished or at risk of malnutrition, and 41% were sarcopenic. Multivariate regression analysis showed lower body mass index (Odds Ratio (OR) = 1.4, 95% CI: 1.1, 1.7, *p* = 0.003) and lower MNA-SF score (OR = 1.6, 95% CI: 1.0, 2.4, *p* = 0.047) were predictive of sarcopenia after controlling for age, level of care, depression, and number of medications. Findings highlight the need for regular malnutrition screening in RAC to prevent the development of sarcopenia, where low weight or unintentional weight loss should prompt sarcopenia screening and assessment.

## 1. Introduction

Sarcopenia describes the decline in muscle mass and function that occurs with age [1] and is exacerbated by inadequate nutrient intake, reduced physical movement, inflammation and diseases that increase nutrient requirements or affect the endocrine system [2]. Loss of muscle strength and function is associated with many negative outcomes for older adults, such as a reduced ability for self-care [3] and lower quality of life [4]. Sarcopenia is associated with an increased risk of falls and fractures [5], hospital admissions [6], pneumonia [7], chronic respiratory diseases [8] and all-cause mortality [9]. Among community of older people living in Europe, an association between increased muscle strength and decreased lower-limb reaction times has been observed [10], which may lead to an increased risk of falling.

In 2019, the European Working Group on Sarcopenia in Older People (EWGSOP) criteria for sarcopenia was accepted as the operational definition for use by New Zealand clinicians and researchers [11]. Under these criteria, a positive sarcopenia diagnosis is represented by: poor muscle strength determined by handgrip strength or chair to stand tests, and low muscle quantity or quality, assessed through dual-energy X-ray absorptiometry (DEXA), bioelectrical impedance assessment (BIA), computerised tomography or magnetic resonance imaging technology. If physical performance is deemed to be poor via an appropriate measure, such as gait speed, timed up-and-go or the physical performance battery, then the sarcopenia is considered to be severe [2]. The establishment of a consensus definition for sarcopenia allows for better comparisons of studies as well as increased opportunity for agreement between health professionals, to provide more effective treatment to those diagnosed [11].

While the co-existence of impaired bone health (osteopenia/osteoporosis), sarcopenia and obesity has been described among older people, the need for a consensus on a definition of osteosarcopenic obesity is yet to be determined [12]. 

Studies overseas that have used the EWGSOP criteria to assess residents living in residential aged care (RAC) report varying rates of sarcopenia [13,14,15]. For example, sarcopenia prevalence among RAC residents of 40.2% in Australia [14] and 38.7% in Slovenia [15] has been reported, with a slightly lower prevalence in Italy (32.8%), China (32.5%) and Egypt (17.7%) [13,16,17]. While these studies had small sample sizes of 80 to 277 participants, results suggest that, among residents in RAC, sarcopenia may be a widespread condition. 

Little is known about the prevalence of sarcopenia among RAC residents in New Zealand. Since the EWGSOP operational definition of sarcopenia has only recently been implemented in New Zealand, now is an appropriate time to begin to fill this knowledge gap [11]. Therefore, this study aimed to investigate the prevalence of sarcopenia and associated risk factors among older adults in RAC. 

## 2. Materials and Methods

### 2.1. Study Design and Recruitment

**Figure 1:**
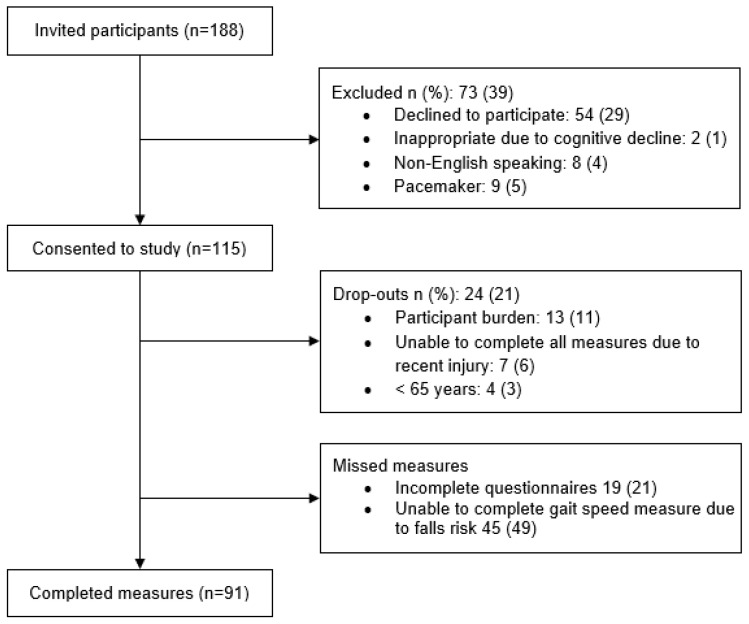
Participant recruitment flow chart.

This cross-sectional study was conducted among older adults living in three RAC facilities in Auckland, operated by a national aged care provider. Eligible residents were aged ≥65 years; residing in rest home or hospital level of care; and able to provide informed consent, or if unable, proxy-informed consent could be obtained from a family member. Exclusion criteria included residents with a pacemaker or those deemed ineligible by the clinical manager based on an acute decline in cognition or function or the need for acute palliative care. Details of participant recruitment are shown in Figure 1. 

Eligible residents or their enduring power of attorney were guided through a Participant Information Sheet and provided written informed consent before data were collected. The study was approved by the New Zealand Health and Disability Ethics Committee 20/NTB/120/AM01.

### 2.2. Data Collection

Data were collected at each facility by researchers trained in all aspects of the assessments. In each facility, participant characteristics, such as age, gender, ethnicity, highest level of education, marital status, length of stay, level of care, prescribed medications, comorbidities and fall history, were recorded from the rest home’s clinical notes. Face-to-face personal interviews were then undertaken with each participant using validated questionnaires, and responses were collected on electronic tablets. 

#### 2.2.1. Questionnaires

##### SARC-F

The Strength, Assistance with walking, Rising from a chair, Climbing stairs, and experiencing Falls (SARC-F) questionnaire was used to identify those at increased risk of sarcopenia and is a five-item screening tool for sarcopenia recommended for use in the EWGSOP diagnostic criteria [2]. Each question is scored between 0 and 2, with a score of ≥4 indicating risk of sarcopenia and requiring further assessment [2]. 

##### Malnutrition

Malnutrition risk was identified using the Mini Nutrition Assessment–Short Form (MNA-SF), a six-item questionnaire validated for geriatrics across a range of settings [18]. The MNA-SF considers food intake, weight loss and physical or psychological stress over the last three months, as well as body mass index (BMI) [19]. There are three possible classifications from the MNA-SF scoring, 0–7 points (malnourished), 8–11 points (at risk of malnutrition), or 12–14 points (normal nutrition status) [18]. For participants unable to answer the MNA-SF questions due to cognitive decline, the participant’s carer provided responses on their behalf. 

##### Dysphagia

Dysphagia risk was assessed using the validated Eating Assessment Tool (EAT-10), a 10-item self-directed questionnaire [20]. Each question identifies how much of a problem swallowing is in a variety of circumstances; each question is rated from zero to four, with zero being no problem and four being a severe problem. A score of three or more is classified as being abnormal, with higher scores indicating more severe dysphagia [20].

##### Depression

Depression was measured using the 15-item Geriatric Depression Scale (GDS-15) designed as a screening tool in the older population and validated with a specificity of 95% and sensitivity of 84% [21,22]. Each question has equal weighting and a value of zero or one; a total score of five or more is suggestive of depression [23].

##### Quality of Life

The Medical Outcomes Study 12-item Short Form Survey (SF-12) tool was used to inform physical and mental quality of life. The SF-12 considers physical functioning, pain and energy levels, social functioning and mental and physical health and produces two summary results: a physical and a mental component summary score [24]. The SF-12 has been validated for the older adult population, with significant correlations between physical and mental health and number of chronic illnesses [25].

#### 2.2.2. Physical Measures

##### Height and Weight

Height (cm) was recorded to the nearest 0.1 cm using a portable stadiometer (model 213; SECA GmbH, Hamburg, Germany). For participants who were chair-bound or bed-bound, ulna length was measured to the nearest 0.5 cm and validated equations were used to predict height [26]. Ulna length was chosen over demi-span, as it was an easier measure to complete for those with cognitive decline. Weight (kg) was taken to the nearest 0.1 kg using a portable, calibrated scale (model 813; SECA, Germany). The rest home’s calibrated chair hoist was used to measure the weight of non-weight bearing participants to the nearest 0.1 kg. 

##### Grip Strength

Grip strength (kg) was measured to the nearest 0.1 kg using a JAMAR hydraulic hand dynamometer (model #5030J1; Patterson Medical, Warrenville, IL, USA). Participants were seated and held the hand dynamometer keeping the forearm at a 90-degree angle. Participants completed the measure three times on each hand and the highest of the six measures was recorded to the nearest kilogram. The EGWSOP cut-off point of 27 kg for men and 16 kg for women was used to indicate low muscle strength [2]. 

##### Gait Speed

Physical functioning was assessed with a 2.4-m walking test. A cone was placed in a clear space with even flooring, a piece of tape was placed to mark 0.6 m after the cone, a second piece of tape was used to mark 2.4 m from the first piece of tape and a cone was placed 0.6 m after the final piece of tape. The participant was asked to walk at their normal pace between the two cones. Using a stopwatch (model 46-139; HART Sport, Shenzen, China), a researcher timed the walk between the two taped marks and recorded the time to 0.1 s. The timed walk was complete three times; the time was then converted into metres per second (m/s) speed by dividing 2.4 by the time taken. The fastest of the three 2.4-m walk times was then converted into a 4-m gait speed using the following conversion equations [27]:

For 2.4-m gait speed ≤ 1.0 m/s: 4-m gait speed = 0.01 + (2.4-m gait speed × 1.052)

For 2.4-m gait speed > 1.0 m/s: 4-m gait speed = 0.481 + (2.4-m gait speed × 0.581)

The EWGSOP cut-off point of 0.8 m/s was used to indicate poor physical function. Note was taken of any walking aids used to complete the measure. Those at a high risk of falls as determined by the RAC clinical manager or who were chair bound were given a score comparative to those who walked slower than 0.8 m/s for the gait speed test. 

##### Body Composition

Participants’ body composition was measured using the Inbody S10 bioelectrical impedance scales (InBody Co., Ltd., Seoul, Korea). Appendicular muscle mass, body fat percentage, fat mass, bone mineral composition, skeletal muscle mass and fat free mass were collected. In Body S10 is a validated method for estimating skeletal muscle mass compared with dual-energy X-ray absorptiometry, the gold standard measure [28]. BIA measurements were taken with participants in a supine position either in a reclined armchair or on the participant’s bed with arms placed away from the body and legs separated. Participants who had a pacemaker, implanted cardioverter-defibrillator or injuries requiring bandages at the site of the electrodes were excluded from this measure. Muscle mass was measured, and appendicular muscle mass/height^2^ was compared to the EWGSOP cut-off points for low muscle mass of <7 kg/m^2^ for men and <5.5 kg/m^2^ for women [2]. 

##### Sarcopenia

Sarcopenia was diagnosed according to the EWGSOP guidelines [2]; a positive diagnosis was determined when both grip strength measured with a hand dynamometer, and appendicular muscle mass/height^2^ measured with BIA, were below the EWGSOP sex specific cut-off points. Sarcopenia was deemed severe if the individual’s gait speed was also slower than 0.8 m/s.

### 2.3. Statistical Analysis

Statistical analyses were carried out using SPSS statistical software (Version 27, SPSS Inc., Chicago, IL, USA). Continuous variables for population characteristics were assessed for normality using the Shapiro–Wilk and Kolmogorov–Smirnov tests. The log of non-normal data was taken, and normality was re-checked. Parametric data were presented as mean and standard deviation, and non-parametric data as medians with 25th and 75th percentiles. Categorical variables were presented as frequencies and percentages. The association between factors and positive sarcopenia diagnoses were assessed using independent sample *t*-tests for parametric data and Mann–Whitney U tests for non-parametric data. Chi-squared tests were used to compare categorical variables. Predictors of sarcopenia were assessed employing binary logistic regression analysis (univariable and multivariable). Six independent variables with strong associations with sarcopenia were entered into the regression model. Despite being significantly associated with sarcopenia in the preliminary cross-tabulation analysis, diabetes and cancer were not included in the regression due to the small number of people with these conditions. To avoid the violation of multicollinearity and incomplete information from the predictors due to many variables with many categories and because there was a strong relationship between BMI and body fat% (BF%) (r = 0.7, *X*^2^ (1) =19, *p* < 0.0001), separate binary logistic regression analyses were performed, with either BMI or BF%. Imbalanced data with binary outcome variables are associated with biases in the estimated probability of an event. The models were investigated to determine if all the assumptions were met, and which model had a better model fit. The regression model containing BMI had a better goodness of fit and was better at predicting sarcopenia, with a sensitivity of 83.3% and specificity of 90.7%. This was compared with the BF% regression model that had a sensitivity of 79.2% and specificity 83.7%. Because of this, the regression model containing BMI was favoured over the model with BF% for the regression analysis. Interaction terms were added into the models to investigate interaction effects between variables, but no significant results were observed. Associations were described using adjusted odds ratios (OR) and 95% confidence intervals (CI). Statistical significance for all statistical tests was determined at *p* < 0.05.

## 3. Results

### 3.1. Sarcopenia Prevalence and Participant Characteristics

A total of 37 (41%) participants were sarcopenic using the EWGSOP criteria. Sarcopenic individuals were older (*p* = 0.01) and more likely to be receiving hospital rather than rest home level of care (*p* = 0.005). The characteristics of the participants are provided in Table 1. Sarcopenic individuals were less likely to be taking more than seven medications (*p* = 0.004) and more likely to have diabetes (*p* = 0.03) or a malignancy (*p* = 0.05) than non-sarcopenic participants (Table 2). 

### 3.2. Body Composition

The association between anthropometric measurements, body composition, grip strength, gait speed and sarcopenia status are presented in Table 3. As all sarcopenic individuals walked slower than the EWGSOP cut-off for poor physical function (<0.8 m/s) they were all considered as severe cases. Only seven participants had a walking speed faster than 0.8 m/s and they were not sarcopenic. Of those who completed this measure, 75.6% of participants used a walking aid such as a frame or stick.

### 3.3. Participants’ Nutritional Status and Mental/Physical Well-Being

Those with sarcopenia were more likely to have a GDS-15 score indicative of depressive symptoms (*p* = 0.006) (Table 2).

**Figure 2:**
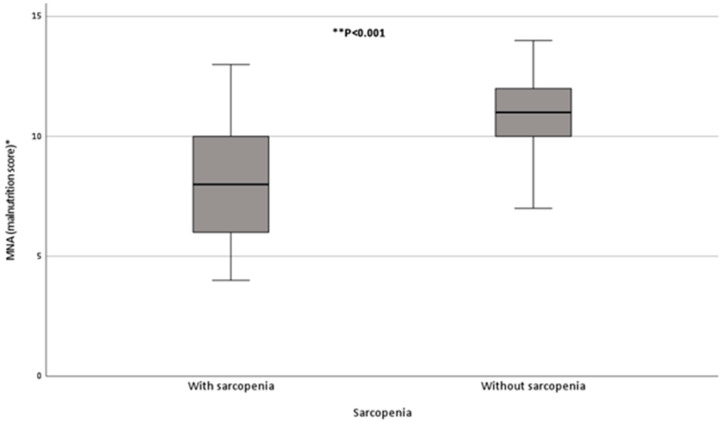
Malnutrition score (assessed by MNA-SF) among participants with and without sarcopenia. Participants with Sarcopenia had lower median (25th, 75th percentiles) MNA-SF score than those without sarcopenia, 8 (6, 10) vs. 11 (10, 12) units, *p* < 0.001. * Higher scores are indicative of better nutritional status. ** Significant at *p* < 0.05.

Most (83%) of the participants had MNA-SF scores indicative of malnutrition (26%) or nutrition risk (63%) (Table 2). Participants with sarcopenia were significantly more likely to be malnourished or at risk of malnutrition (*p* = 0.004) (Table 2). Participants with sarcopenia had significantly lower median (25th, 75th percentiles) MNA-SF scores than those without sarcopenia, 8 (6, 10) vs. 11 (10, 12) units, respectively (*p* < 0.001) (Figure 2).

### 3.4. Factors Predicting Sarcopenia 

Odds ratios for all variables included in the multivariate logistic regression containing BMI and the regression model containing BF% are presented in Table 4 and Table 5, respectively. Hospital level of care, depressive symptoms, lower BMI, MNA-SF score and increasing age were significantly associated with sarcopenia in the univariate analysis (*p* < 0.05). After controlling for confounding variables, only BMI (OR: 1.4; 95% CI: 1.1–1.7) and MNA-SF score (OR: 1.6; 95% CI: 1.0, 2.4) remained significant predictors of sarcopenia in the regression containing BMI. MNA-SF score was also found to be predictive of sarcopenia in the regression analysis containing BF% (OR: 1.6; 95% CI: 1.1, 2.4). Whilst age was not a significant predictor of sarcopenia in the regression model containing BMI (OR = 0.9, 95% CI: 0.8–1.0), it was marginally significant in the regression model where BF% was used (OR = 1.1, 95% CI: 0.8–1.0).

## 4. Discussion

Using the EWGSOP diagnostic criteria [2], this study among RAC residents found sarcopenia prevalence to be 41%, with all cases deemed to be severe. Those with sarcopenia tended to be older (mean age ± SD; 88.6 ± 7.6 years) than those without sarcopenia (mean age ± SD; 84.2 ± 8.4 years). Sarcopenia affected many RAC residents in the sample surveyed, and while this study sample is not representative of the New Zealand population, our findings provide evidence to suggest sarcopenia may be prevalent in New Zealand RAC. While participants completed the recommended screening questionnaire, SARC-F, scores were not significantly different between sarcopenic and non-sarcopenic participants. This supports current evidence that the self-reported SARC-F may have moderate to low sensitivity [29] and is probably indicative of some degree of cognitive impairment among the participants.

Multivariate analysis identified decreasing BMI and decreasing MNA-SF score to be significant predictors of increasing sarcopenic risk among the participants, which highlights the importance of regular nutrition screening and treatment of malnutrition in RAC. Sarcopenia and malnutrition often overlap, with symptoms and drivers of each independent condition being tightly intertwined [30]. Muscle wasting occurs as a direct consequence of malnutrition, as adequate protein and energy intake are required for the prevention of muscle protein breakdown [31,32,33]. In the current study, most (83%) residents were malnourished or at nutrition risk, which is similar to the 90% malnutrition risk reported among 174 New Zealand older adults newly admitted to RAC [34]. The position statement on undernutrition by the Australian and New Zealand Society for Geriatric Medicine [35] suggests all older people be screened and assessed for undernutrition and sarcopenia on a regular basis. Screening is necessary to prompt early intervention and to prevent the progression of malnutrition and sarcopenia with adverse health outcomes [36,37]. The association between decreasing BMI and increasing sarcopenia risk is consistent with findings from previous studies, and further emphasises the need for malnutrition screening [13,14]. Six sarcopenic individuals in the current study had a BMI >25 kg/m^2^, with one individual having a BMI >30 kg/m^2^. Sarcopenia existing alongside obesity has been previously described, with a prevalence between 4% and 12% among community-dwelling older adults (aged ≥60 years) in America [38,39] and Italy [40]. Among hospitalised older people in Europe, sarcopenic obesity was found to be prevalent in 8% of women and 22% of men and in contrast to subjects with sarcopenia (12% of women and 23% of men) had a better nutritional status and metabolic profile [41]. Sarcopenia obesity in older adults is however associated with higher physical disability and mortality rates than sarcopenia alone [42,43]. Therefore, sarcopenia screening is recommended for all RAC residents regardless of the individual’s BMI. In studies that have used the EWGSOP diagnostic criteria, the prevalence of sarcopenia in RAC residents has been reported between 17.7% and 40.2% [14,15,16,17], with variations in prevalence rates likely due to geographical, cultural, or ethnic differences. For example, among residents in Egypt, a lower rate of sarcopenia (17.7%) was attributed to increased sunlight hours, as vitamin D status is well known to be protective of muscle mass in older adults [16]. A study analysing sarcopenia across older adults living in four RAC facilities in Chengdu City, China (mean age ± SD; 81.6 ± 3.3 years) found sarcopenia prevalence to be 32.5% when assessed using the EWGSOP criteria [17]. This is similar to the prevalence of 32.8% found in an Italian study that took place in RAC facilities in Rome which also used the EWGSOP criteria (age ± SD; 84.1 ± 4.8 years) [13]. Many studies report that Asian ethnicity typically has lower muscle mass than Caucasians [44,45], however, the differences in sarcopenia rates in those living in RAC do not appear to reflect this. The current evidence available on sarcopenia in RAC residents suggests it may be a prevalent issue regardless of culture, country, or ethnicity. 

Low hand grip strength among participants with sarcopenia is a key risk factor for functional decrease and loss of independence as it is associated with reduced lower-limb strength [46]. As increased muscle strength is associated with decreased reaction times in older people [10], protective responses to reduce the risk of falls may be ineffective due to lack of muscle strength and movement speed [47]. 

We found that diabetes, as well as malignancies, was significantly associated with sarcopenia, although the small sample size meant that neither of these conditions could be included in the regression model. Diabetes has previously been associated with low muscle mass and strength among community-dwelling older adults [48]. Hyperglycaemia and diabetic neuropathy can impair muscle function and contribute to atrophy, leading to sarcopenia [49,50]. Sarcopenic individuals also have a reduced ability to oxidise glucose through muscle tissue, thus contributing to insulin resistance and the development of diabetes [49]. The cascade of metabolic abnormalities is due to the combination of visceral fat and muscle loss [12] leading to further adverse health effects. 

Sarcopenia prevalence among those with malignancies is reported to range from 11–74% in all adults, with prevalence often higher in older populations [51]. Cancer cachexia causes metabolic alterations, such as decreased appetite, and increased energy requirements and inflammation, which result in muscle wasting [52]. Medications, surgeries and increased bed rest due to cancer can further perpetuate muscle wasting [53]. 

There are several limitations in this study. The small sample size and mean age of the participants limits generalisability. BIA measurements were not undertaken on participants with pacemakers or injuries requiring bandages on the hands or feet where electrodes were placed, which further limited the sample size. While all questionnaires used in this study were validated for the older adult population, some of the residents in this study were cognitively impaired. This does challenge the validity of responses to the questionnaires, for example, responses to the GDS-15 questionnaire have been shown to deteriorate when working with residents with cognitive decline [54]. While the results of this study inform the sarcopenia prevalence of the participants surveyed, the cross-sectional design of this study means causation of sarcopenia cannot be derived from the results. 

## 5. Conclusions

This study is the first to assess the prevalence and risk factors of sarcopenia using the EWGSOP criteria within the New Zealand RAC setting. Our study found that sarcopenia and malnutrition rates were high among participants from three RAC facilities. Sarcopenia was associated with higher scores for malnutrition and lower BMI. The relationship between malnutrition score and sarcopenia provides further rationale to support regular malnutrition screening in RAC and highlights the importance of optimising nutrition to prevent loss of body weight among older adults.

## Figures and Tables

**Table 1:** Characteristics of the participants by sarcopenia status.

	Total (*n* = 91)	Sarcopenic *n* (%): 37 (41)	Non-Sarcopenic *n* (%): 54 (59)	*p*-Value *
Age, years, mean ± SD	86.0 ± 8.3	88.6 ± 7.6	84.2 ± 8.4	0.01 *
Age, years, *n* (%)				0.14
<85	38 (42)	12 (32)	26 (68)	
≥85	53 (58)	25 (47)	28 (53)	
Gender, *n* (%)				0.80
Women	58 (64)	23 (40)	35 (60)	
Men	33 (36)	14 (42)	19 (58)	
Ethnicity (*n* = 88) ^1^, *n* (%)				0.84
New Zealand European	59 (67)	23 (39)	36 (61)	
Other ^2^	29 (33)	13 (45)	16 (55)	
Marital status (*n* = 76) ^1^, *n* (%)				0.79
Partnered	29 (38)	12 (41)	17 (59	
No partner	47 (62)	18 (38)	29 (62)	
Level of education (*n* = 72) ^1^, *n* (%)				0.48
Less than tertiary	25 (35)	8 (32)	17 (68)	
Tertiary and higher	47 (65)	19 (40)	28 (60)	
Length of stay (*n* = 76) ^1^, *n* (%)				0.15
≤30 months	48 (63)	16 (33)	32 (67)	
>30 months	28 (37)	14 (50)	14 (50)	
Level of care (*n* = 91), *n* (%)				0.005 *
Rest home level	53 (58)	15 (28)	38 (72)	
Hospital level	38 (42)	22 (58)	16 (42)	
Oral nutritional supplement use (*n* = 85) ^1^, *n* (%)				0.52
Yes	18 (21)	6 (33)	12 (67)	
No	67 (79)	28 (42)	39 (58)	

* Chi-square test; significant difference between sarcopenic and non-sarcopenic participants (*p* < 0.05). Abbreviations: SD, standard deviation. ^1^ Missing data for variable. ^2^ Other ethnicities: Māori, Fijian Indian, Chinese, South African, European, Australian, Fijian.

**Table 2:** The association of sarcopenia with medication use and co-morbidities.

	Total (*n* = 91)	Sarcopenic *n* (%): 37 (41)	Non-Sarcopenic *n* (%): 54 (59)	*p*-Value *
Number of regular medications (*n* = 81), *n* (%) ^1^				0.004 *
≤7	41 (51)	23 (56)	18 (44)	
>7	40 (49)	10 (25)	30 (75)	
Comorbidities (*n* = 86), *n* (%) ^1^				
Number of comorbidities				0.90
≤5	41 (48)	17 (42)	24 (58)	
>5	45 (52)	18 (40)	27 (60)	
Hypertension				0.22
No	45 (54)	16 (36)	29 (64)	
Yes	39 (46)	19 (49)	20 (51)	
Cardiovascular diseases				0.94
No	26 (31)	11 (42)	15 (58)	
Yes	59 (69)	24 (41)	34 (59)	
Diabetes				0.03 *
No	71 (79)	26 (37)	45 (63)	
Yes	13 (21)	9 (69)	4 (31)	
Cognitive impairment				0.66
No	48 (57)	19 (40)	29 (60)	
Yes	36 (43)	16 (44)	20 (56)	
Renal diseases				0.35
No	68 (81)	30 (44)	38 (56)	
Yes	16 (19)	5 (31)	11 (69)	
Cancer				0.05 *
No	74 (88)	28 (38)	46 (62)	
Yes	10 (12)	7 (70)	3 (30)	
Chronic respiratory diseases				0.72
No	71 (85)	29 (41)	42 (59)	
Yes	13 (15)	6 (46)	7 (54)	
Arthritis				0.23
No	64 (76)	29 (45)	35 (55)	
Yes	20 (24)	6 (30)	14 (70)	
Fracture				0.91
No	74 (88)	31 (42)	43 (58)	
Yes	10 (12)	4 (40)	6 (60)	
SARC-F Score (*n* = 76) ^1^				
<4	35 (46)	10 (29)	25 (71)	0.34
≥4	41 (54)	16 (39)	25 (61)	
Dysphagia (*n* = 76) ^1^				0.19
Not at risk	53 (70)	17 (32)	36 (68)	
At-risk	23 (30)	11 (48)	12 (52)	
Depression (*n* = 72) ^1^				0.006 *
Low risk	47 (65)	11 (23)	36 (77)	
At-risk/high risk	25 (35)	14 (56)	11 (44)	
Malnutrition (*n* = 87) ^1^				0.004
Not at risk	15 (17)	1 (7)	14 (93)	
At-risk/malnourished	72 (83)	34 (47)	38 (53)	
SF-12 Physical Component Score (*n* = 61) ^1^				0.73
≥50	48 (80)	19 (86)	29 (76)	
<50	12 (20)	3 (14)	9 (24)	
SF-12 Mental Component Score(*n* = 61) ^1^				0.36
≥42	10 (17)	5 (23)	5 (13)	
<42	50 (83)	17 (77)	33 (87)	

* Chi-square test; significant at *p* < 0.05. Abbreviations: SF-12, Medical Outcomes Study’s 12 item Short Form Survey. ^1^ Missing data for variable.

**Table 3:** The association of sarcopenia with anthropometric, body composition and strength/function measures.

	Total (*n* = 91)	Sarcopenic *n* (%): 37 (41)	Non-Sarcopenic *n* (%): 54 (59)	*p*-Value *
BMI (kg/m^2^)	24.9 ± 6.1	21.6 ± 3.7	27.7 ± 5.9	<0.001 *
Fat mass, mean ± SD (kg)	25 ± 12	19 ± 8	30 ± 12	<0.001 *
BF%, mean ± SD (kg)	37 ± 11	33 ± 9.7	39 ± 11	0.01 *
Fat free mass (kg)	40 (34, 47)	34 (31, 40)	42 (37, 51)	<0.001 *
Skeletal mass index (kg/h^2^)	6.1 (5.3, 7.1)	5.1 (4.7, 5.8)	6.8 (6.0, 7.7)	<0.001 *
Skeletal muscle mass (kg)	20 (17, 25)	17 (15, 21)	22 (20, 27)	<0.001 *
Appendicular lean mass (kg)	15 (13, 20)	13 (11, 15)	17 (15, 22)	<0.001 *
Bone mineral content (kg)	2.4 (2.1, 2.8)	2.3 (2.0, 2.6)	2.5 (2.3, 2.9)	0.003 *
Grip strength, mean ± SD (kg) (*n* = 82) ^1^	13.9 ± 7.8	9.5 ± 5.9	16.7 ± 7.6	<0.001 *
Gait speed, mean ± SD (m/s) (*n* = 46) ^1^	0.55 ± 1.42	0.49 ± 1.30	0.57 ± 1.45	0.175

* Independent sample *t*-test for normally distributed data and Mann–Whitney U test for not normally distributed data. Significant at *p* < 0.05. Abbreviations: BMI, body mass index; BF%, body fat percentage; h, height in cm; SD, standard deviation. Values are reported as median (25th, 75th percentiles) unless otherwise stated. ^1^ Missing data for variable.

**Table 4:** Factors predicting sarcopenia using a regression model including BMI.

	Total	With Sarcopenia	Without Sarcopenia	OR (95% CI)	
				Univariate	Multivariate
Malnutrition score ^1^	9.5 ± 2.3	8.2 ± 2.1	10.0 ± 1.9	1.7 (1.3, 2.2)	1.6 (1.0, 2.4)
Depression score ^2^	4.4 ± 3.3	5.4 ± 3.8	3.7 ± 2.9	0.9 (0.7, 1.0)	0.8 (0.6, 1.1)
BMI (kg/m^2^)	24.9 ± 6.1	21.6 ± 3.7	27.7 ± 5.9	1.4 (1.2, 1.6)	1.4 (1.1, 1.7)
Age (years)	86.0 ± 8.3	88.6 ± 7.6	84.2 ± 8.8	0.9 (0.9, 1.0)	0.9 (0.8, 1.0)
Number of regular medications	7.7 ± 3.4	6.4 ± 3.0	8.7 ± 3.4	1.3 (1.0, 1.5)	1.1 (0.8, 1.4)
Level of care, *n* (%)					
Rest home care	53 (58)	15 (28)	38 (72)	Reference category	
Hospital care	38 (42)	22 (58)	16 (42)	3.5 (1.4, 8.4)	1.0 (0.2, 5.6)

Abbreviations: BMI, body mass index; OR, odds datio; CI, confidence interval. Values reported as mean ± SD unless otherwise stated. ^1^ Assessed by Mini Nutrition Assessment Short-Form (MNA-SF). ^2^ Assessed by Geriatric Depression Scale 15-item questionnaire (GDS-15).

**Table 5:** Factors predicting sarcopenia using regression model containing BF%.

	Total	With Sarcopenia	Without Sarcopenia	OR (95% CI)	
				Univariate	Multivariate
Malnutrition score ^1^	9.5 ± 2.3	8.2 ± 2.1	10 ± 1.9	1.7 (1.3, 2.2)	1.6 (1.1, 2.4)
Depression score ^2^	4.4 ± 3.3	5.4 ± 3.8	3.7 ± 2.9	0.9 (0.7, 1.0)	0.8 (0.6, 1.0)
BF%	37 ± 11	33 ± 10	39 ± 11	1.1 (1.0, 1.1)	1.1 (1.0, 1.1)
Age (years)	86.0 ± 8.3	88.6 ± 7.6	84.2 ± 8.8	0.9 (0.9, 1.0)	0.9 (0.8, 1.0)
Number of regular medications	7.7 ± 3.4	6.4 ± 3.0	8.7 ± 3.4	1.3 (1.0, 1.5)	1.2 (0.9, 1.5)
Level of care, *n* (%)					
Rest home care	53 (58)	15 (28)	38 (72)	Reference category	
Hospital care	38 (42)	22 (58)	16 (42)	3.5 (1.4, 8.4)	1.0 (0.2, 4.7)

Abbreviations: BF%, body fat percentage; OR, odds ratio; CI, confidence interval. Values reported as mean ± SD unless otherwise stated. ^1^ Assessed by Mini Nutrition Assessment Short-Form (MNA-SF). ^2^ Assessed by Geriatric Depression Scale 15-item questionnaire (GDS-15).

## Data Availability

The data presented in this are available upon request from the corresponding author.
